# Comparative Evaluation of Three Preprocessing Methods for Extraction and Detection of Influenza A Virus Nucleic Acids from Sputum

**DOI:** 10.3389/fmed.2018.00056

**Published:** 2018-03-02

**Authors:** Fei Yu, Ting Qiu, Ying Zeng, Yiyin Wang, Shufa Zheng, Xiao Chen, Yu Chen

**Affiliations:** ^1^Key Laboratory of Clinical In Vitro Diagnostic Techniques of Zhejiang Province, Department of Clinical Laboratory, First Affiliated Hospital, College of Medicine, Zhejiang University, Hangzhou, China; ^2^School of Laboratory Medicine and Life Science, Wenzhou Medical University, Wenzhou, China

**Keywords:** sputum, homogenization, nucleic acid, purity, concentration, influenza A virus

## Abstract

Viscous sputum specimens usually cannot undergo automated extraction, and thus, a pre-homogenization process is desirable before isolating nucleic acids for real-time reverse transcription PCR. In this study, we compared three preprocessing methods [preprocessing with normal saline (NS), dithiothreitol (DTT), and proteinase K (PK)] of sputum specimens on the extraction and detection of influenza A virus (IAV) nucleic acids. Based on the experimental results of 217 specimens, we found that DTT and PK could be used to improve the homogenization effects of sputum and increase the positive rates by 5.53–6.91% higher than that of the NS group. Comparison of 49 positive specimens in all of the three groups demonstrated that the threshold cycle values of the DTT group and PK group were significantly lower and their nucleic acid concentration and *A*_260_/*A*_280_ ratio within 1.8–2.0 were higher than those of the NS group. Thus, sputum homogenization before nucleic acid extraction is essential for the accurate diagnosis of IAV infection.

## Introduction

Human infection with avian influenza A (H7N9) has been remaining persistent in China over the recent years. For laboratory diagnosis of H7N9 virus infection, related studies and guidelines have confirmed that the nucleic acid-positive rate of lower respiratory specimens, such as sputum, airway aspirates, and bronchoalveolar lavage fluid, is higher than that of upper respiratory specimens (1, 2). Clinicians give preference to sputum specimen for influenza virus test and genotyping because this kind of specimen is easy to obtain. However, real-time reverse transcription PCR (rRT-PCR) of sputum specimens yields false-negative results owing to the difficulty of extracting RNA from sputum containing mucus (3). Meanwhile, viscous sputum specimens usually cannot undergo automated extraction, and thus, a pre-homogenization process is desirable before isolating nucleic acids for rRT-PCR. In fact, some influenza A virus (IAV) detection kits lack homogenization reagents, and some clinical laboratories may have applied improper preprocessing methods, such as adding normal saline (NS) and mixing by vortex.

Proteinase K (PK), a common component in the nucleic acid isolation kit of blood specimens, is a serine protease that exhibits broad cleavage specificity (4) and degrades RNases in samples, resulting in prevention of RNA degradation (3). The sulfhydryl reagent dithiothreitol (DTT), a superior reagent to reduce mucoprotein disulfide bonds specifically and completely, is widely used for sputum sample homogenization (5–7). Therefore, in this study, we compared these three preprocessing methods of sputum specimens on the extraction and detection of IAV nucleic acids to provide a basis for the laboratory preprocessing of sputum specimens.

## Materials and Methods

### Specimen Source

The sputum specimens of IAV test were received from the Department of Clinical Laboratory, First Affiliated Hospital, College of Medicine, Zhejiang University from January7th to February 18th, 2017. A total of 626 IAV test specimens, including 373 sputum specimens (59.6%), were collected in 43 days. And 217 sputum specimens over 1.5 ml were used for the comparative test in this study. The study was approved by the Ethical Review Board of the First Affiliated Hospital, College of Medicine, Zhejiang University.

### Three Preprocessing Methods

**Figure 1:**
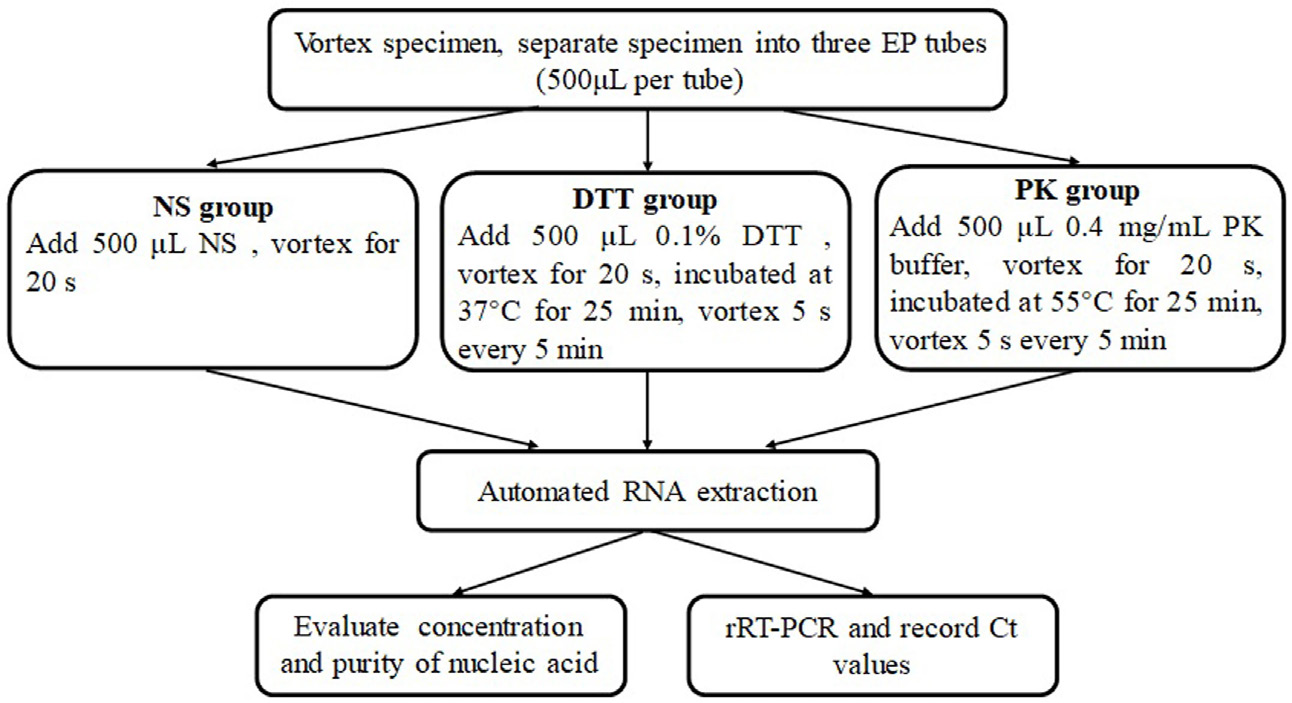
Flowchart of experiment operation in this study.

Each included specimen was collected into three EP tubes separately (500 µl per tube) by using a disposable pipette after vortex. Each tube of specimens was preprocessed with NS, DTT (Sputasol Liquid, France), and PK (Figure 1). Then, 200 µl of specimens was taken to extract nucleic acids.

### RNA Extraction and rRT-PCR Testing

RNA was extracted simultaneously everyday by using the Automated Nucleic Acid Extraction System through a paramagnetic beads method (Zhijiang Biotechnology Co., Ltd., Shanghai, China). The concentration and purity (*A*_260_/*A*_280_ ratio) of nucleic acid for each RNA extract were evaluated with a Nanodrop 2000 spectrophotometer (Thermo Fisher Scientific, Wilmington, DE, USA). *A*_260_/*A*_280_ ratio of pure RNA products ranged from 1.8 to 2.0 (8). Extracted RNA was tested by rRT-PCR using IAV Detection kit (Zhijiang Biotechnology Co., Ltd.) with ABI 7500 Real-Time PCR instrument (Foster City, CA, USA). Threshold cycle (Ct) values being negatively related to concentration of nucleic acid were recorded. Operation and result assessment were conducted in accordance with the manufacturers’ instructions. Qualified negative, positive, and internal controls were the premise of results validity.

### Statistical Analysis

Data were statistically analyzed with Excel 2007 and SPSS 18.0. The Ct values among the three groups were compared through one-way ANOVA. Comparison between two groups was tested by LSD. The measurement data of abnormal distribution were described by median (M) and quartile (P_25_–P_75_) and subsequently evaluated through nonparametric Kruskal–Wallis test. *P* < 0.05 indicated significant differences.

## Results

### Test Results

Of the 217 sputum specimens, 66 (30.41%) were positive in at least one group. 49 (22.58%), 64 (29.49%), and 61 (28.11%) were positive in the NS, DTT, and PK groups, respectively. 17 (25.76%), 2 (3.03%), and 5 (7.58%) positive specimens were missed in the NS, DTT, and PK groups, respectively.

### Comparison of Positive Specimens in all of the Three Groups

**Figure 2:**
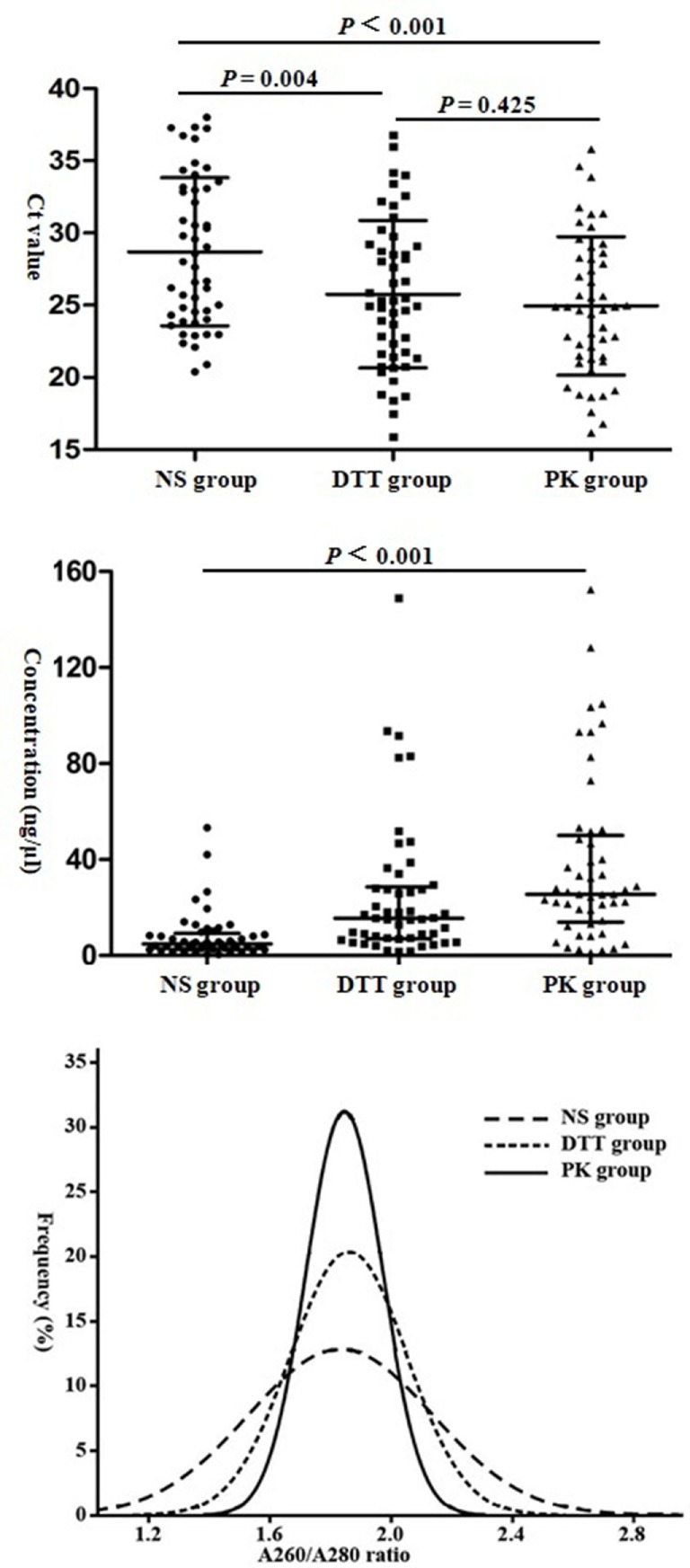
Comparison of threshold cycle (Ct) value $\left(\bar{x}\pm s\right)$, nucleic acid concentration (median and P_25_–P_75_), and purity of 49 positive specimens in all three groups. Abbreviations: NS group, normal saline group; DTT, dithiothreitol group; PK group, proteinase K group.

A total of 49 specimens were tested positive in all of the three groups. The mean Ct values of the NS, DTT, and PK groups were 28.70 ± 5.12, 25.76 ± 5.11, and 24.95 ± 4.79, respectively, with statistically significant difference (*P* = 0.001) (Figure 2). The M of nucleic acid concentrations (ng/μl) in the three groups were 4.80 (P_25_–P_75_: 2.90–9.25), 15.50 (P_25_–P_75_: 7.05–28.65), and 25.50 (P_25_–P_75_: 13.95–50.10), respectively, with *P* < 0.001 among the three groups. The mean *A*_260_/*A*_280_ ratios of the NS, DTT, and PK groups were 1.84 ± 0.31, 1.86 ± 0.19, and 1.85 ± 0.13, respectively. The proportions of *A*_260_/*A*_280_ ratios within the range of 1.8–2.0 were 30.61, 57.14, and 75.51%, respectively.

### Specimens with Inconsistent Results among the Three Groups

Of the 217 sputum specimens, 17 specimens yielded inconsistent results in the three preprocessing methods, all of which were tested negative in the NS group. Among these specimens, 10 were positive in both DTT and PK groups, 5 were positive in the DTT group but negative in the PK group, and 2 were positive in the PK group but negative in the DTT group. The mean Ct values of the positive results in the DTT and PK groups were 33.89 ± 1.86 and 33.18 ± 2.81, respectively. The M of nucleic acid concentration in the NS, DTT, and PK groups were 3.80 (P_25_–P_75_: 1.60–9.35), 13.40 (P_25_–P_75_: 4.35–24.15), and 24.10 (P_25_–P_75_: 7.00–55.95), respectively. The proportions of *A*_260_/*A*_280_ ratios within 1.8–2.0 were 41.18, 64.71, and 64.71%, respectively (Table 1).

**Table 1:** Threshold cycle (Ct) value, nucleic acid concentration, and purity of 17 specimens with inconsistent test results in three groups.

Sample no.	Normal saline group			Dithiothreitol group			Proteinase K group		
	Ct value	Concentration (ng/μl)	*A*_260_/*A*_280_ ratio	Ct value	Concentration (ng/μl)	*A*_260_/*A*_280_ ratio	Ct value	Concentration (ng/μl)	*A*_260_/*A*_280_ ratio
20170109MLA019^a^	–	5.4	2.00	32.64	2.4	2.28	31.79	6.7	1.76
20170109MLA021^a^	–	99.7	1.87	34.72	176.3	1.87	34.75	99.0	1.87
20170110MLA011^a^^,^^b^	–	1.0	2.19	31.57	50.0	1.83	28.41	120.4	1.85
20170112MLA009	–	3.8	1.52	–	39.5	1.82	35.7	24.2	1.81
20170112MLA013^a^^,^^b^	–	10.7	1.86	34.44	19.5	1.84	–	24.1	1.79
20170114MLA014^a^^,^^b^	–	1.7	2.52	32.34	13.6	1.90	–	45.0	1.82
20170116MLA009^a^^,^^b^	–	5.8	2.01	35.52	16.4	1.89	–	22.2	1.90
20170119MLA007^a^	–	15.3	1.80	31.94	27.4	1.89	33.46	117.0	1.82
20170120MLA012^a^^,^^b^	–	8.3	1.77	33.32	13.4	1.93	33.02	66.9	1.84
20170122MLA003	–	1.8	2.30	34.77	3.6	2.15	–	13.8	1.79
20170125MLA011^b^	–	6.1	1.66	30.44	10.2	1.89	28.64	30.9	1.83
20170206MLA028^a^^,^^b^	–	1.5	1.99	–	6.4	1.93	30.82	5.6	1.99
20170207MLA012^b^	–	2.2	2.43	34.17	7.2	2.09	34.86	19.6	1.96
20170208MLA002	–	1.4	2.26	35.27	1.3	2.34	34.62	0.3	2.23
20170208MLA007^b^	–	3.1	1.91	33.85	20.9	1.90	34.32	29.8	1.83
20170210MLA008	–	1.3	1.54	36.01	3.8	1.71	–	7.3	1.76
20170218MLA003	–	10.4	1.92	37.41	4.9	2.43	37.77	3.2	2.42

*^a^Genotyped as H7N9 avian influenza virus*.

*^b^Blood-tinged sputum*.

*“–” is undetermined*.

## Discussion

During H7N9 virus epidemic, nearly 60% of the specimens received by laboratories to detect influenza virus were sputum. For these specimens, appropriate preprocess was essential. In this study, all of the sufficient sputum samples were preprocessed in three methods, IAV RNA extracted and tested by the same method, and comparison results were of important guiding significance.

It was reported previously that treatment with *N*-acetyl-l-cysteine and sodium citrate solution came out in whole homogenization of sputum samples, which was unable to destroy mRNA or rRNA of mycobacteria in sputum samples. However, for the purpose of detecting RNA targets in purulent sputum, this method was not appropriate (3, 9). DTT and PK are often used for the homogenization processing of sputum specimens, which can fully digest mucous protein, release epithelial cells, and consequently increase the concentration of extracted RNA. Purulent sputum samples with treatment of DTT or PK can undergo automated extraction. Based on the experimental results of 217 specimens, our conclusion is that DTT and PK could be used to improve the homogenization effects of sputum and increase the positive rates by 5.53–6.91% higher than that of the NS group. Of the 17 missed specimens, 9 were genotyped as H7N9. Although these specimens were weakly positive, the missed detection of these highly pathogenic agents might result in serious consequences (1). Vortex with NS is often adopted in respiratory viruses’ culture from sputum, but the method is not appropriate for detection of IAV by rRT-PCR.

Sufficient nucleic acid extraction and the removal of substances that inhibit amplification are critical factors influencing the detection of IAV by rRT-PCR (10). Comparison of 49 positive specimens in all of the three groups demonstrated that the Ct values of the DTT and PK groups were significantly lower and their nucleic acid concentration and *A*_260_/*A*_280_ ratio within 1.8–2.0 were higher than those of the NS group. This finding might be attributed to the inability of NS to dilute sticky sputum through violent vortex. Some cells cannot be released from sticky sputum and thus lead to inadequate specimen decomposition and RNA extract reduction. Meanwhile, high amounts of proteins in sticky liquid are retained and can influence PCR by disturbing the cell lysis of nucleic acid extraction that inhibits DNA polymerase activity.

Moreover, the proportion for blood-tinged sputum specimens in the 17 specimens yielded inconsistent results of 52.94% (9/17), significantly higher than that of the specimens with consistent results in the three groups (28.00%, 56/200). For few negative specimens, concentration and purity were higher, which was inconsistent with the results. Therefore, the nucleic acid purity and concentration examined with a spectrophotometer were not the only factor that determined the positive rate. These findings indicated that false-negative results might be attributed to PCR inhibitor residues in nucleic acids. Further optimizing of liquefier combinations, preprocessing steps and nucleic acid extraction reagents could be conducted to decrease PCR inhibitors and increase target nucleic acid concentration.

In summary, DTT or PK treatment before nucleic acid extraction is essential for detection of IAV infection from sputum samples. Furthermore, adequate sputum homogenization is worth considering for accurate diagnosis of respiratory viruses infection.

## Author Contributions

YC, XC, and FY designed the study; TQ, YZ, and FY performed the experiments; FY, SZ, and TQ analyzed the data; FY, TQ, and YW wrote the manuscript. All the authors read and approved the final manuscript.

## Conflict of Interest Statement

The authors declare that the research was conducted in the absence of any commercial or financial relationships that could be construed as a potential conflict of interest.
